# The impact of anxiety on affective and cognitive empathy

**DOI:** 10.1371/journal.pone.0315156

**Published:** 2025-11-21

**Authors:** Manjeet Susch, Andrew D. R. Surtees

**Affiliations:** 1 School of Psychology, University of Birmingham, Birmingham, United Kingdom; 2 Birmingham Women’s and Children’s NHS Foundation Trust, Birmingham, United Kingdom; University of Glasgow, UNITED KINGDOM OF GREAT BRITAIN AND NORTHERN IRELAND

## Abstract

Existing evidence about the way anxiety impacts empathy is mixed, highlighting the complexity of empathy as a construct. The impact of state anxiety on affective and cognitive empathy in women was tested. Seventy-five women underwent an anxiety or relaxation induction, prior to completing measures of affective and cognitive empathy, and trait anxiety. Robust positive correlations were found between trait cognitive and trait affective empathy, and trait and performance affective empathy. Induced anxiety impaired affective empathy performance when controlling for trait empathy, however, was not observed to impact cognitive empathy performance. Trait anxiety and empathy did not moderate the influence of induced anxiety on affective empathy performance. Irrespective of trait levels of anxiety and empathy, women are less affectively empathic when anxious, but do not vary in their cognitive empathy.

Empathy is the capacity to share and understand the thoughts and feelings of others and is a crucial element of social interaction, prosocial behaviour and moral development [1]. Empathy enables people to make sense of and predict others’ feelings and behaviours, to then connect and respond accordingly [2]. Empathy does not occur in isolation from other abilities and some individuals are more empathic than others, which relates to their other traits [3,4]. Within individuals, empathy for others can be affected by other internal states, such as anxiety [5,6]. Understanding these relationships is crucial because it is often when we are anxious that understanding others is most important, such as when Emergency Room doctors try to understand the wishes of a person’s family during urgent procedures, or Clinical Psychologists look to understand someone’s feelings and intentions when they report suicidal ideation.

Amongst the numerous conceptualisations of empathy, the notion of two separable components of affective and cognitive empathy is the most accepted [7]. Considerations of affective empathy typically refer to the co-experience of the same emotional state as the other, for instance experiencing personal distress as a result of another person’s unpleasant situation [8]. Affective empathy is characterised by a conscious self-other distinction [9], setting it apart from other processes, such as emotion contagion and mimicry. Cognitive empathy, conversely, refers to the process of recognising and identifying the emotional states of others [7] and incorporates a range of mechanisms, including aspects of perspective-taking and Theory of Mind [10]. Cognitive and affective empathy are postulated as dissociable constructs, associated with differing neurocognitive processes, and deficits resulting in differential outcomes [11]. Early conceptualisations focused on empathy as a measurable, static or trait-like ability, that could not be taught [12]. Later conceptualisations of empathy outlined the presence of a dynamic variability within individuals, namely state empathy [13]. Fluctuations in mood and psychological states have been shown to cause within-person variability in empathy [14].

There is increasing interest in the impact that anxiety has on empathy. Anxiety is a negative valence, high arousal emotion, that is characterised by the experience of uncertainty [15]. Anxiety is a multi-dimensional construct. Trait, anxiety reflects an individual’s tendency to experience anxiety across contexts, and is related to internal behaviours, such as apprehension and worry [16]. State anxiety is an unpleasant reaction accompanied by physiological arousal, in response to a potential threat [17]. Frequent and severe experiences of state anxiety, behaviours associated with high levels of trait anxiety and an impact on functioning form core criteria for anxiety disorders [18]. Differences across disorders reflect differences in symptoms, but also meaningful differences in anxiety that occurs across domains, for instance between social and more generalized experience of anxiety.

Increased social sensitivity and awareness of others’ distressing situations may be related to the experience of anxiety [19]. Anxiety may result in increased worry for others in the form of compassion and greater consideration of how one may impact the other, and thus increase empathic ability [19]. Uncertainty remains regarding whether affective responses, such as anxiety, are characteristic of or obstacles to the empathy process [20]. The existing literature, however, largely focuses on the experience of social anxiety (for a review see: [6]), or anxiety within therapists and the impact on therapeutic relationships [21].

It is not necessarily the case that anxiety will have the same impact on empathy for everyone. Gender differences in trait empathy are widely discussed, and it is generally accepted that women have greater trait empathy [3,22]. The mediating influence of gender during state-like empathy processes is also widely discussed. Tomova et al. [23] reported that when subjected to acute levels of stress, women demonstrated increased self-other distinction ability and reduced emotional egocentricity bias. Lower emotional egocentricity bias led to faster, more affiliative responses towards a target other, that were less likely to be influenced by their own emotional states [23]. The authors highlighted that when stressed, women were able to respond flexibly from self or other perspectives depending on the task demands [23], consistent with increased cognitive empathy. Conversely, when under stress, men’s egocentric responses increased. The ‘tend-and-befriend’ hypothesis is often provided as an explanation for the increased ability of empathy of women during periods of heightened stress [24]. This hypothesis posits that when exposed to threat, women will tend to offspring to promote their survival and seek affiliation with others for joint comfort and protection [24]. Whilst studies have reported that women are able to recognize emotional expressions faster and more accurately than men (e.g., [25]), it is argued that trait empathy is often not taken into consideration [26]. Martínez-Velázquez et al. [26] reported that higher trait empathy was associated with greater affective and cognitive empathic responses in both men and women but did not appear to influence emotional recognition ability.

Understanding the impact of anxiety on empathy is crucial to understanding how we interact with one another in the most pressured environments. Existing evidence presents a confused picture, through which anxiety may be associated with either less or more empathy for others [27,28]. One reason for this confusion may be potentially different relationships with cognitive versus affective empathy. Another reason may be a lack of appreciation of trait-like empathy differences between individuals. Sex differences are apparent in anxiety [29], perspective-taking processes in social anxiety [23] and self-reported empathy [30] – meaning focussing on single sex samples is important. This study explored: i) The relationship between trait measures of anxiety, cognitive empathy and affective empathy, ii) The impact of state anxiety on cognitive and affective empathy performance, iii) How the impact of state anxiety is moderated by trait anxiety and empathy.

## Method

### Participants

Seventy-five participants, with a mean age of 21.4 years, were recruited for the study in accordance with the inclusion criteria: self-identified gender and sex as female and not having diagnoses of neurodevelopmental disorder (e.g., Learning Disability or Autism Spectrum Disorder), or acute or chronic psychiatric illness requiring secondary care. All participants were over the age of 18. Participants were selected using a convenience sampling method and were either students (*n = *49) recruited through the University of Birmingham or members of the public, (*n* = 26, mean age = 25.5) recruited through social media. Participants received study credits or £5 Amazon vouchers for participation. Participants were pseudo-randomly assigned to the Anxiety condition *(n* = 38, mean age = 21.7) or the Relaxation condition *(n* = 37, mean age = 21.2). Participants were recruited between 10^th^ November 2020 and 6^th^ April 2021 and gave written informed consent.

### Sample size rationale

As stated at pre-registration, the original target sample size was 90 participants. A power analysis was conducted using G*Power to identify the key effect of anxiety group on empathy performance through an ANCOVA, including trait empathy and trait anxiety as covariates. To identify a large effect size (*f* = .4), with power = 0.95, at *p* < .05, required a total sample size of 84. The smaller final sample size reflected limitations on the timeframe of a doctoral research project. This was determined as appropriate for drawing meaningful conclusions, as using the same parameters suggested 75 participants would still provide power of.93 to detect an effect of the same size. We acknowledge, however an increased risk of type-2 error and lower power to detect smaller effects.

### Procedure

Ethical approval was obtained through the university’s ethics committee, and the study was pre-registered with the Open Science Framework (see: https://osf.io/pndyw). Participants provided informed consent and initially completed questionnaires over Qualtrics and then direct testing via *Zoom* videoconferencing.

#### Questionnaires.

The Penn State Worry Questionnaire (PSWQ) [31] is widely used as a measure of worry and generalised anxiety disorder (GAD). The PSWQ is a 16-item measure consisting of 11 items pertaining to the experience of pathological worry and five reverse-scored items relating to worry not being a problem. Responses are made using a five-point Likert scale from 1 (‘not at all typical of me’) to 5 (‘very typical of me’). The PSWQ has good test-retest [31] and internal reliability [32] (Chronbach’s alphas ranging from.88 to.95) in undergraduate students and community samples. The measure has high convergent validity with other measures of worry, anxiety and trait anxiety [33] and has demonstrated a high discriminant validity [31].

The Questionnaire of Cognitive and Affective Empathy *(*QCAE) [34] provides separate scales of cognitive and affective empathy. The 31-item questionnaire was developed by amalgamating items rated to have the highest face-validity from existing validated self-report measures, such as the Empathy Quotient [35] and the Interpersonal Reactivity Index (IRI) [4]. The development process of the QCAE included rigorous psychometric analyses in a large sample of undergraduate students and employees, which revealed high reliability and strong convergent and construct validity [34]. The five subscales were identified through exploratory factor analysis during the development of the measure and indicated high internal validity [34].

#### Mood induction.

Participants were alternately assigned to one of two conditions (Anxiety or Relaxation). Checks were conducted after every 20 participants to ensure that there were no significant differences between the groups on self-reported trait empathy or anxiety scores. When significant differences were identified, parity was restored through assigning participants based on a median split of the relevant measure, before pseudo-random assignment was resumed.

Participants engaged in mood induction (anxiety or relaxation) at the beginning, halfway through the cognitive empathy paradigm and before completing the emotional empathy paradigm. Mood induction in the experimental condition was based on instructions adapted from Cataldo & Cohen [36] and involved participants recalling and writing about a time they felt “very anxious” for five minutes. Participants were explicitly asked not to choose a memory of a social nature and shared their screens to allow the experimenter to check that they had followed instructions. Autobiographical memory tasks and writing about emotionally evocative experiences are shown to be effective ways of experimentally inducing target emotions [37]. Mood induction in the Relaxation condition involved a five-minute Progressive Muscle Relaxation (PMR) exercise. Mood induction ‘top-ups’ were provided to ensure that the effect of the target emotion was sustained throughout the study. Participants in the experimental condition were asked to continue writing about their experience of anxiety for the ‘top-up’ and engage with the PMR exercise in the Relaxation condition. Following mood induction and ‘top-ups’, participants rated their current experience of seven emotions (including anxiety and relaxation) using a 9-point Likert scale. The mean rating was calculated for each participant’s scores of self-reported anxiety and relaxation collected across the three mood manipulation checks.

#### Empathy performance measures.

All participants completed measures of Cognitive Empathy (the Reading the Mind in the Eyes Task- RMET) and Affective Empathy (the Pictorial Empathy Test- PET). The order of the completion of study tasks (i.e., the RMET and the PET) was counterbalanced, whereby some participants completed the RMET first whilst others completed the PET and then the RMET.

The Reading the Mind in the Eyes Test (RMET) [38] is considered a valid and reliable measure of social cognition and emotional and social processing [39]. Participants were presented with a series of 36 photographs depicting the eyes along with four words from which participants were asked to choose one that best matched what they felt the person in the photograph was thinking or feeling. Two versions of the RMET were administered (counterbalanced across participants) and items were either presented in the original or reverse order. Due to its length, the measure was administered in two halves with a mood induction top-up in the middle.

The Pictorial Empathy *Test* (PET) [11] aims to provide a brief measure of momentary reactions of affective empathy and has good psychometric properties; the PET is reported to have high reliability and validity [11]. The PET entails presenting seven pictures depicting individuals in distress and asking participants to rate the extent to which they experience the photograph as ‘emotionally moving’. Participants provided their rating using a Likert scale (1 = not at all- 5 = very much). The total score of the PET is obtained by calculating the mean score of seven responses.

### Data analysis

Initially, data were checked using individual samples t-tests to examine whether there were any differences between Anxiety and Relaxation groups on trait anxiety, trait cognitive empathy, trait affective empathy, or age. Following this, a similar t-test was conducted to examine whether the manipulation was successful, by comparing self-reported anxiety across the two groups.

The relationships between trait variables were examined by conducting correlations. As data for some measures were not normally distributed (see Supplementary Materials), Spearman’s Rank correlations were conducted to test whether self-reported Trait Empathy measures were correlated to State Empathy performance. For correlations between empathy measures, 1-tailed tests were undertaken (as an inverse correlation between two theoretically overlapping constructs would be hard to interpret as meaningful). For correlations between empathy measures and anxiety, 2-tailed tests were undertaken. A Bonferroni correction was employed to control for multiple comparisons.

The initial analysis plan that was pre-registered (see: https://osf.io/pndyw) included statistical analysis using ANCOVA as a method of determining the impact of state anxiety on state empathy whilst controlling for trait anxiety and empathy. As requirements of parametric ANCOVA were not met by the data (see S1 File), all ANCOVA were calculated using bootstrap estimates for means, standard errors, and confidence intervals. As the statistical reliability was calculated using bootstrap resampling, with 1000 resamples, these analyses are robust to violation of parametric assumptions.

## Results

### Trait comparison between groups

There were no significant differences in self-reported Trait Anxiety (*t*(73) =.288, *p = *.774), Affective Empathy (*t*(73) =.592, *p *= .556), and Cognitive Empathy (*t*(73) = −.117, *p* = .907), between participants in the Anxiety and Relaxation conditions. There was no significant difference (*t*(73) =.495, *p* = .622), between the mean ages of participants in the Anxiety (*M* = 21.66) and Relaxation groups (*M* = 21.22).

### Manipulation check

Participants reported significantly higher experience of Anxiety in the Anxiety condition (*t*(73) = 6.890, *p *< .005), and a significantly higher experience of Relaxation in the Relaxation condition (*t*(73) = −7.811, *p *< .005).

### Relationship between trait measures

The correlation matrix in Table 1 shows that there were several significant correlations, which were upheld when applying a Bonferroni correction of (*p *< 0.005): Self-reported Trait Affective and Trait Cognitive Empathy were positively correlated. Additionally, Performance Affective Empathy was significantly positively correlated with self-reported Affective Empathy.

**Table 1:** Correlations (Spearman’s Rho (p value)) between trait anxiety, trait empathy measures and state empathy measures.

	PSWQ	QCAE (Cognitive)	QCAE (Affective)	RMET	PET
**PSWQ** **(Trait Anxiety)**	**–**				
**QCAE** **(Trait Cognitive Empathy)**	.213 (.067)	–			
**QCAE** **(Trait Affective Empathy)**	.302^†^ (.008)	.360** (.001)	–		
**RMET** **(State Cognitive Empathy)**	−.260^†^ (.025)	.121 (.151)	−.115 (.163)	–	
**PET** **(State Affective Empathy)**	.220 (.058)	.225* (.026)	.333** (.002)	−.144 (.109)	–

* *Correlation is significant at the 0.05 level (1-tailed).*

** Correlation is significant at the 0.005 level (1-tailed).

† *Correlation is significant at the 0.05 level (2-tailed).*

†† *Correlation is significant at the 0.005 level (2-tailed).*

There was some evidence for marginal correlations between other variables, not upheld when applying stringent Bonferroni correction, however, were significant at the *p *< 0.05 level: positive correlations between self-reported Trait Anxiety and self-reported Trait Affective Empathy and between self-reported Cognitive Empathy and Performance Affective Empathy, and a negative correlation between self-reported Trait Anxiety and Performance Cognitive Empathy. Scatterplots for all correlations are included in the S1 File.

### The impact of state anxiety on empathy performance

It was hypothesised that state anxiety would impact cognitive and affective empathy performance. Further, it was hypothesised that trait anxiety would moderate the impact of state anxiety on affective and cognitive empathy processes.

A one-way ANCOVA showed a significant difference in State Affective Empathy scores between Anxiety and Relaxation groups when controlling for Trait Anxiety and self-reported Trait Affective Empathy, *F*(1, 71) = 5.502, *p* = 0.022, *η*_*p*_^*2*^ = .072. Affective Empathy scores were higher in the Relaxation (Mean = 3.89, SD = .58) than the Anxiety condition (Mean = 3.60, SD = .58). Trait Affective Empathy contributed significantly as a covariate in the final model, *F*(1, 71) = 15.539, *p* < .001, *η*_*p*_^*2*^ = .18, but Trait Anxiety did not, *F*(1, 71) =.221, *p* = 0.640, *η*_*p*_^*2*^ = .003.

No significant difference was observed in State Cognitive Empathy scores between Anxiety (Mean = 26.58, SD = 4.41) and Relaxation (Mean = 27.27, SD = 4.00) groups when controlling for Trait Anxiety and self-reported Trait Cognitive Empathy (*F*(1, 71) = 0.386, *p *= 0.536), *η*_*p*_^*2*^ = .005. Trait Anxiety contributed significantly as a covariate in the final model, *F*(1, 71) = 8.578, *p* = .005, *η*_*p*_^*2*^ = .108, but Trait Cognitive Empathy did not, *F*(1, 71) = 2.693, *p = .*105, *η*_*p*_^*2*^ = .037.

### The moderating role of traits on the impact of anxiety empathy performance

The ANCOVA revealed that state anxiety significantly impacted Affective Empathy performance. A regression was conducted to explore the interactions between Group (state Anxiety or Relaxation), Trait Empathy and Anxiety. These interaction terms demonstrate the degree to which the impact of group was modelled consistently across different levels of the trait variable. Assumptions for regression analysis were met (see S1 File). The overall regression analysis was significant, *F*(6, 74) = 3.897, *p *= .002, and accounted for 19% variance (Adjusted *R*^2^ = .190). The only significant contributor to the model was Group (Anxiety or Relaxation), see Table 2. Trait Affective Empathy only evidenced a marginal contribution, which may be partly due to overlap with interaction terms. That the interaction terms did not significantly contribute suggests that the impact of group was consistent across different levels of Trait Empathy and Trait Anxiety. The model therefore predicts that irrespective of trait levels of anxiety and empathy, state anxiety impaired affective empathy ability equally in all women.

**Table 2:** Regression model predicting affective empathy with interaction terms.

Variable	*B*	*SE B*	*β*	*Sig.*
Group (Anxiety/Relaxation)	.330	.146	.242	.027
Trait Anxiety	.009	.010	.147	.346
Trait Affective Empathy	.048	.025	.376	.062
Group x Trait affective empathy	.012	.031	.075	.710
Group x Trait Anxiety	−.013	.014	−.136	.380
Group x Trait Anxiety x Affective Empathy	.000056	.001	.005	.967

## Discussion

This study investigated the relationship between state and trait anxiety and empathy across cognitive and affective domains.

### State anxiety, affective and cognitive empathy performance

Women in the relaxation condition provided significantly higher ratings of affective empathy compared to those in the anxiety condition, when controlling for self-reported trait anxiety and empathy scores: after accounting for trait levels of anxiety and affective empathy, the experience of state anxiety impaired affective empathy. The impact of state anxiety on affective empathy performance was not moderated by levels of self-reported trait anxiety and empathy. This suggests that regardless of underlying empathy or anxiety traits, all women’s affective empathy was impeded by anxiety.

Anxiety resulted in women being less affectively empathic but was not seen to impact their cognitive empathy. Whilst a relatively small effect, the differential impact of anxiety on the separate components of empathy may provide support for the notion of the two distinct empathic reactions of compassion and empathic distress [40]. Anxiety may cause reduced affective empathy due to the experience of empathic distress; the experience of anxiety may lead to a reduced capacity to feel the emotions of others, and a desire to withdraw to protect oneself from negative emotions [41]. Contrarily, cognitive empathy performance appeared to be unaffected by the experience of state anxiety. The differential impact of state anxiety on performance affective and cognitive empathy may be due to the need for complex cognitive skills in cognitive empathy, with affective empathy being a largely automatic process [42].

Alternatively, the differential impact of anxiety on affective vs. cognitive empathy may be to do with how cognitive and affective empathy are conceptualised and measured. Whilst both require a representation of another’s emotions, only affective empathy considers the additional impact of this on the empathiser. Anxiety has been shown to impact processes required for effective emotion regulation, including identification and labelling of emotions in the self [43]. Our measure of affective empathy relied on participants rating how “emotionally moved” they had felt by pictures [11]. One possibility is that anxiety impacted the participants’ ability to identify and report their own emotions, rather than their ability to be affected by the other’s experience as such. Either alternative explanation of the effect has important consequences – recognising one’s empathy may be crucial to acting on it, but future multi-modal research will be needed to differentiate alternatives, such as measuring empathy through neuro-imaging [44] or electrocardiogram [45].

### Relationships between traits and empathy performance

Relationships were reported between the subscales within the cognitive and affective empathy scales [34], supporting the notion that whilst affective and cognitive empathy are related, they remain two distinct constructs. Martínez-Velázquez et al. [30] reported that higher trait empathy was associated with greater affective and cognitive empathic responses in a mixed gender sample. Discrepancies in findings may be due to the documented influence of gender on empathy and the use of an all-female sample in the current study [3].

There was some evidence that self-reported trait anxiety was positively associated with trait affective empathy – though the correlation fell marginally short of significance via a conservative Bonferroni correction. The possible association between trait anxiety and self-reported trait affective empathy may be due to the ‘tend-and-befriend’ phenomenon [46], a desire to associate and affiliate amongst women when anxious.

### Limitations and future directions

The findings of this study are restricted by the limits of the literature and measurement tools available for experimental use. Affective empathy requires the experience of a state that matches that of the other [47] and participants are not required to share the emotion they experience in self-reported measures, such as the PET. It is therefore possible that participants experiencing a high level of mis-matched emotions may receive a score that indicates high affective empathy, inconsistent with the current definition of empathy [47]. Whilst autobiographical memory tasks are widely used and shown to be effective methods of experimentally inducing target emotions in the literature [36] and in the current study, the anxiety induced in this study may be a mild analogue to real-world experiences. Further research exploring how differing levels of anxiety moderate the relationship between anxiety and empathy and replicating the study in a male sample would also be advantageous

Participants were selected using a convenience sampling method, and more detailed information about demographic characteristics was not collected, due to practical limitations. It is important to hold this in mind when considering the generalisability of the study’s results. In addition, the results of this study are based upon lab-based measures, which allow for greater control when measuring and studying relationships between constructs. This degree of control, however, inherently limits the richness of real-life settings and thus the ecological validity of the findings.

## Conclusion

The current study builds upon previous research and provides a novel insight into how anxiety may impact various components of empathy differentially amongst women. The findings suggest the existence of a simple relationship between state anxiety and empathy performance in women; women regardless of their trait levels of anxiety and empathy became less affectively empathic when anxious. A negative correlation was observed between trait anxiety and cognitive empathy performance; however, state anxiety did not appear to influence cognitive empathy performance and was not moderated by trait anxiety and empathy levels. This finding provides insight into an interesting and underexplored phenomenon; suggesting that whilst women may experience a decreased sharing of affect, their ability to understand the emotions of others when they are anxious remains unaffected.

## Supporting information

S1 File Additional information supporting statistical analysis. (DOCX)
