# Supplementary material for: The impact of anxiety on affective and cognitive empathy
Source: PLoS One. 2025 Nov 21;20(11):e0315156. doi: 10.1371/journal.pone.0315156 (PMC12637922; doi:10.1371/journal.pone.0315156)
Supplement: S1 File — (DOCX) [file pone.0315156.s001.docx]

# Supplementary Materials

## Violation in Parametric Assumptions for Correlational Data

Assumption: Normal distribution

| **Tests of Normality** | | | | | | |
| --- | --- | --- | --- | --- | --- | --- |
|  | Kolmogorov-Smirnov^a^ | | | Shapiro-Wilk | | |
|  | Statistic | df | Sig. | Statistic | df | Sig. |
| PSWQ | .054 | 75 | .200^*^ | .988 | 75 | .688 |
| COGEMP | .071 | 75 | .200^*^ | .964 | 75 | .031 |
| Affemp | .102 | 75 | .052 | .967 | 75 | .048 |
| RMETscore | .122 | 75 | .008 | .954 | 75 | .008 |
| PETscore | .134 | 75 | .002 | .936 | 75 | .001 |
| *. This is a lower bound of the true significance. | | | | | | |
| a. Lilliefors Significance Correction | | | | | | |

### ***Assumption: Outliers***


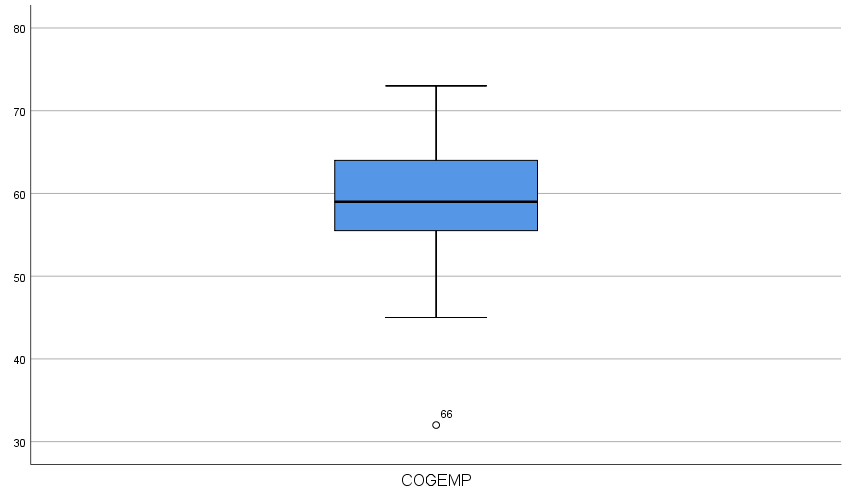


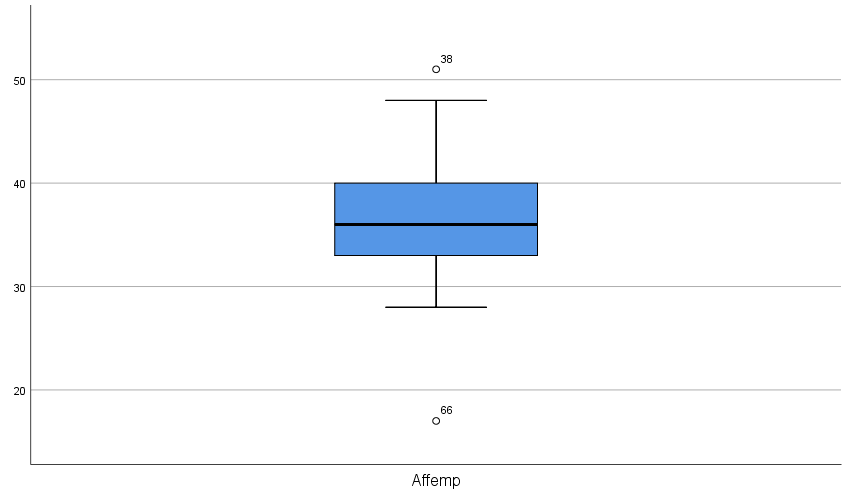


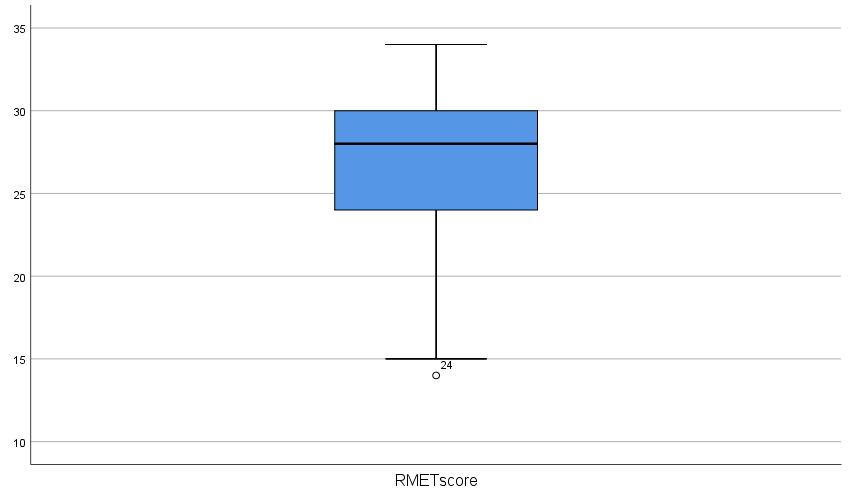


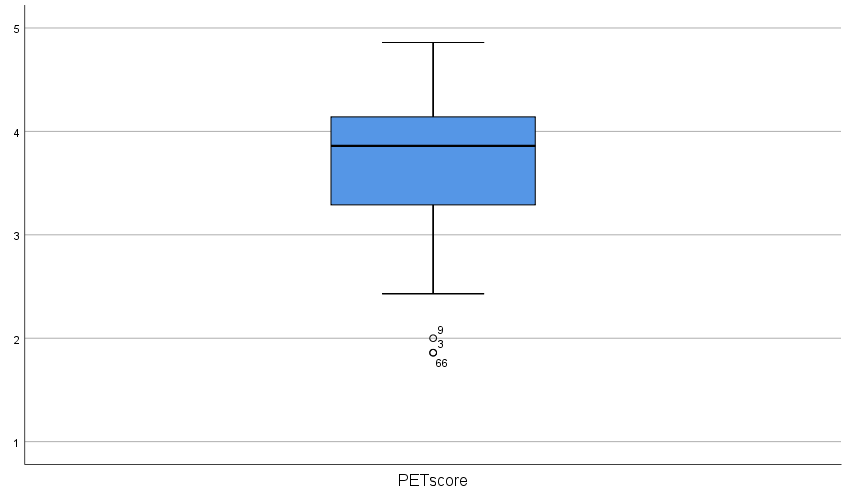


## Scatter Plots for Correlations

***Correlations between Trait Variables***

*
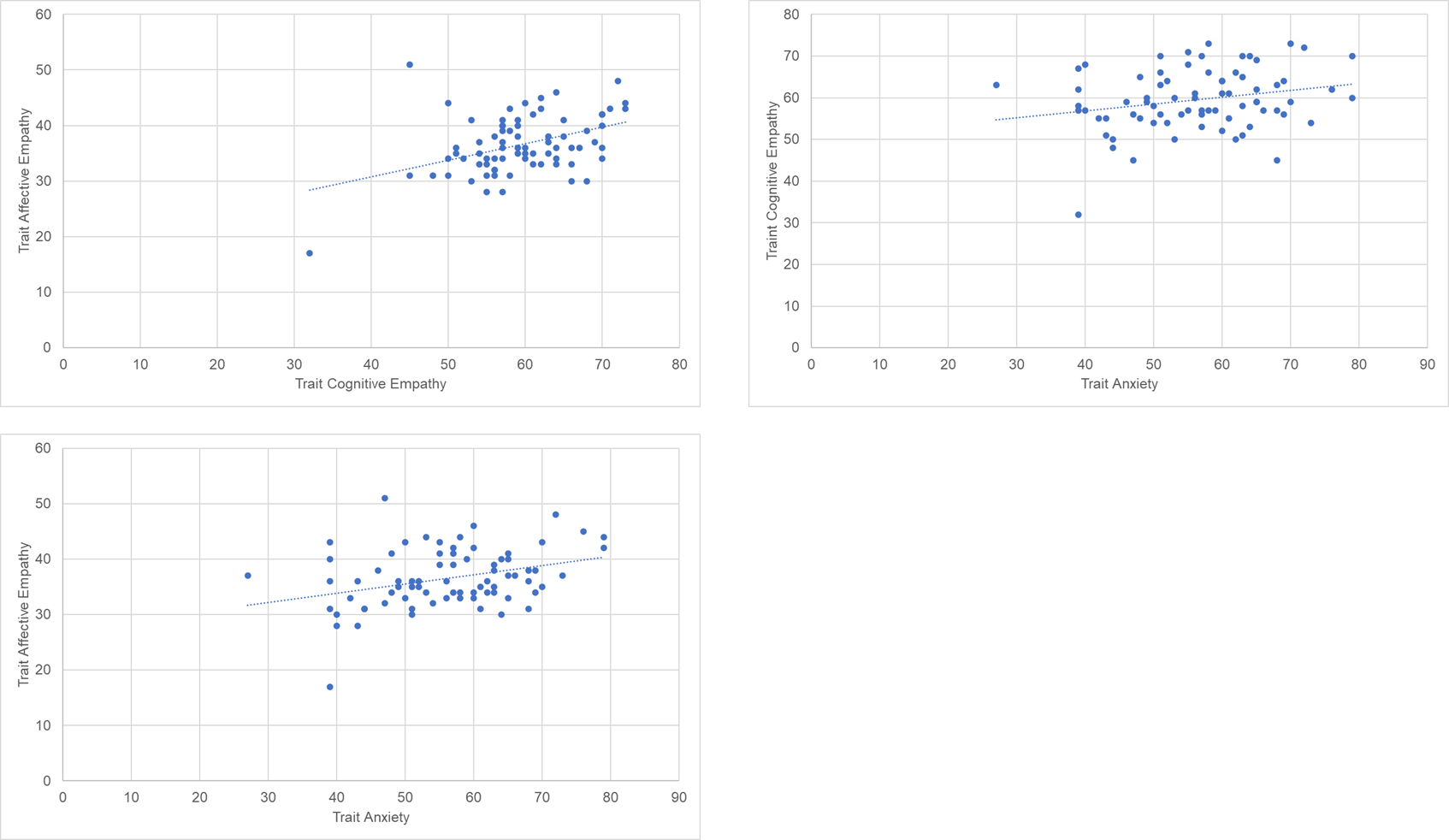
*

***Correlations between Trait Variables and Reading the Mind in the Eyes Task (RMET)***

***
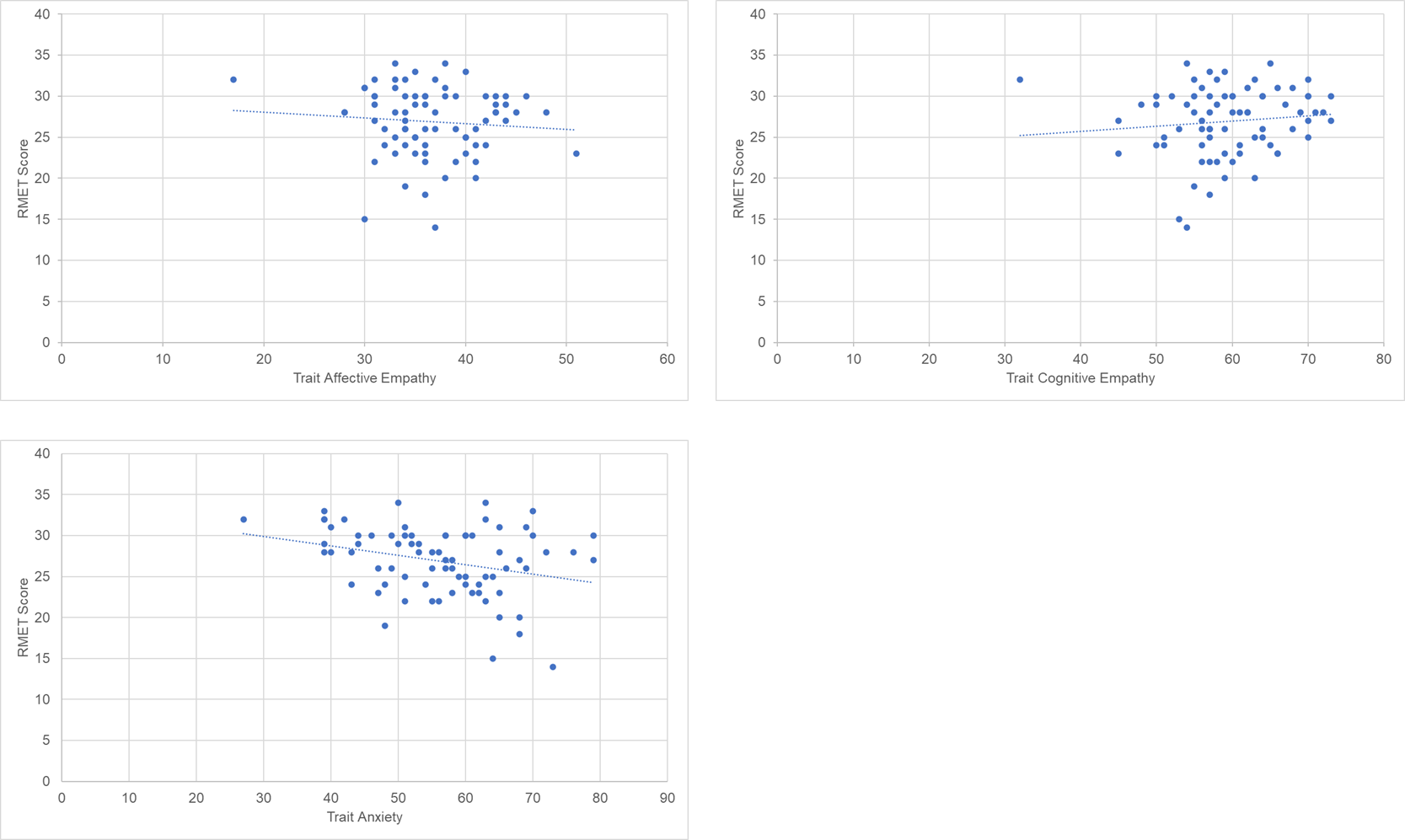
***

***Correlations between Pictorial Empathy Test (PET) Score and other Variables***

***
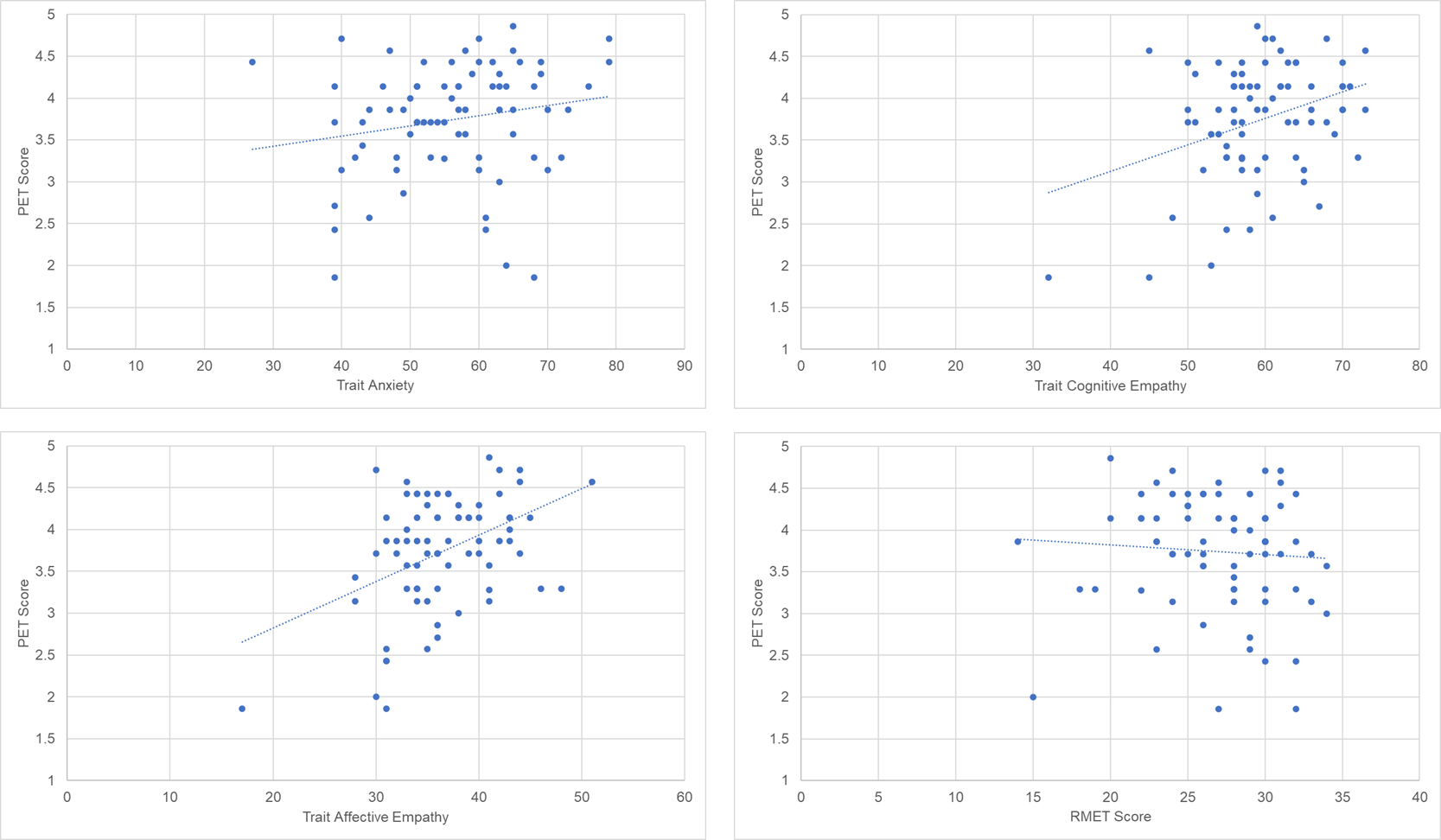
***

## Violation in Parametric Assumption for ANCOVA

### ***Assumption 1: Normal Distribution***

| **Tests of Normality** | | | | | | | |
| --- | --- | --- | --- | --- | --- | --- | --- |
|  | Anx1Rel2 | Kolmogorov-Smirnov^a^ | | | Shapiro-Wilk | | |
|  |  | Statistic | df | Sig. | Statistic | df | Sig. |
| RMETscore | 1.00 | .123 | 38 | .154 | .954 | 38 | .124 |
|  | 2.00 | .140 | 37 | .065 | .942 | 37 | .052 |
| PETscore | 1.00 | .113 | 38 | .200^*^ | .941 | 38 | .047 |
|  | 2.00 | .208 | 37 | .000 | .881 | 37 | .001 |
| *. This is a lower bound of the true significance. | | | | | | | |
| a. Lilliefors Significance Correction | | | | | | | |

### ***Assumption 2: Outliers***


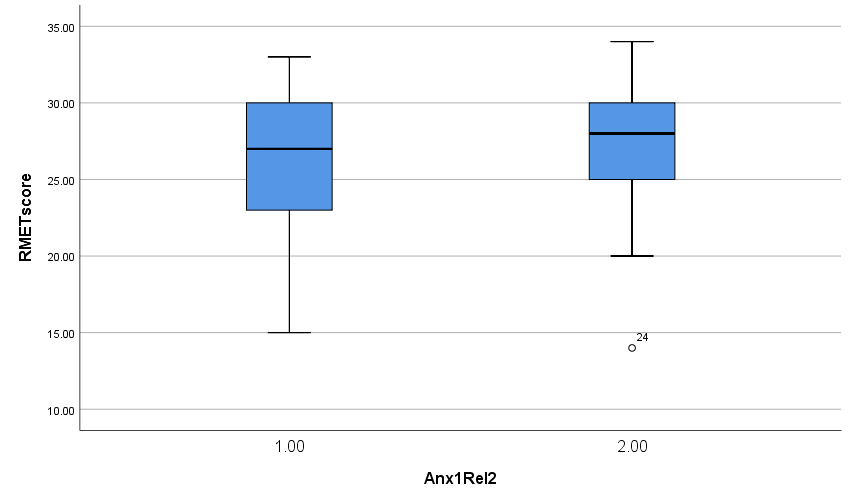


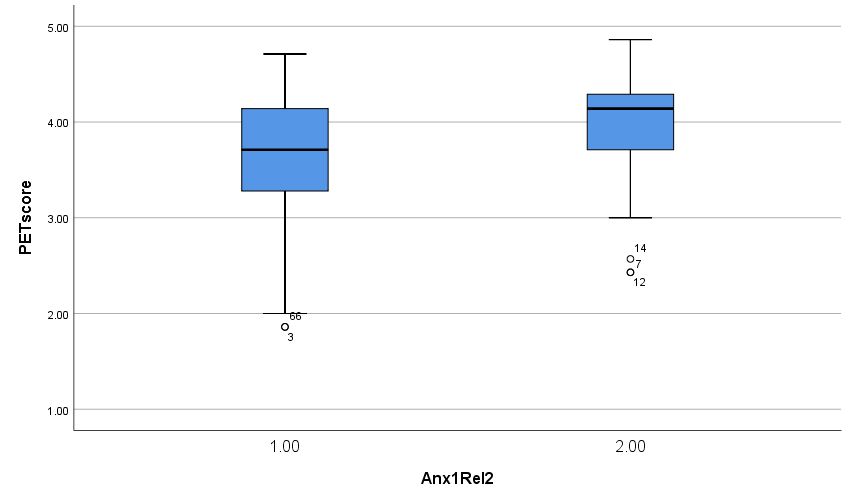


### ***Assumption 3: Linear Relationship between the Covariate and the Dependent Variable for each Level of the Independent Variable***


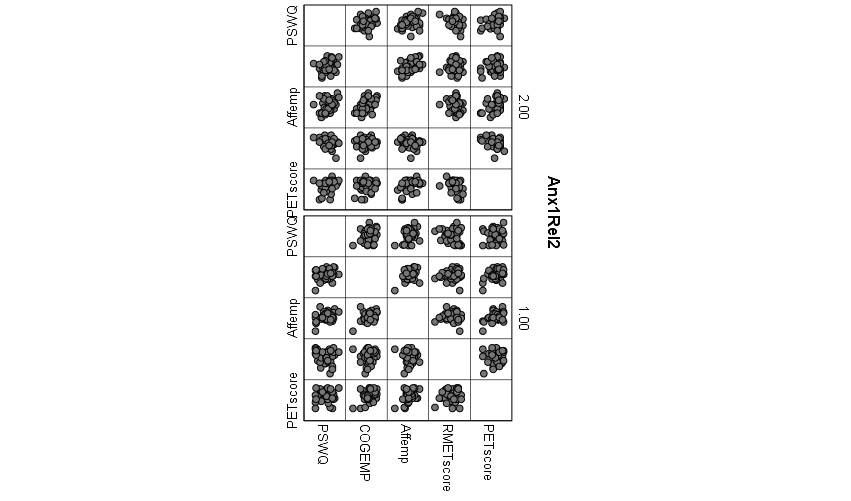


### ***Assumption 4: Homogeneity of Regression Slopes***

| **Tests of Between-Subjects Effects** | | | | | | |
| --- | --- | --- | --- | --- | --- | --- |
| Dependent Variable: RMETscore | | | | | | |
| Source | Type III Sum of Squares | df | Mean Square | F | Sig. | Partial Eta Squared |
| Corrected Model | 18.873^a^ | 2 | 9.437 | .529 | .592 | .014 |
| Intercept | 5948.815 | 1 | 5948.815 | 333.410 | .000 | .822 |
| Anx1Rel2 * PSWQ * Affemp * COGEMP | 18.873 | 2 | 9.437 | .529 | .592 | .014 |
| Error | 1284.647 | 72 | 17.842 |  |  |  |
| Total | 55655.000 | 75 |  |  |  |  |
| Corrected Total | 1303.520 | 74 |  |  |  |  |
| a. R Squared = .014 (Adjusted R Squared = -.013) | | | | | | |

| **Tests of Between-Subjects Effects** | | | | | | |
| --- | --- | --- | --- | --- | --- | --- |
| Dependent Variable: PETscore | | | | | | |
| Source | Type III Sum of Squares | df | Mean Square | F | Sig. | Partial Eta Squared |
| Corrected Model | 5.969^a^ | 2 | 2.985 | 7.459 | .001 | .172 |
| Intercept | 65.340 | 1 | 65.340 | 163.291 | .000 | .694 |
| Anx1Rel2 * PSWQ * Affemp * COGEMP | 5.969 | 2 | 2.985 | 7.459 | .001 | .172 |
| Error | 28.810 | 72 | .400 |  |  |  |
| Total | 1085.271 | 75 |  |  |  |  |
| Corrected Total | 34.779 | 74 |  |  |  |  |
| a. R Squared = .172 (Adjusted R Squared = .149) | | | | | | |

### ***Assumption 5: Homogeneity of Variance***

| **Levene's Test of Equality of Error Variances^a^** | | | |
| --- | --- | --- | --- |
| Dependent Variable: RMETscore | | | |
| F | df1 | df2 | Sig. |
| 1.921 | 1 | 73 | .170 |
| Tests the null hypothesis that the error variance of the dependent variable is equal across groups. | | | |
| a. Design: Intercept + PSWQ + Affemp + COGEMP + Anx1Rel2 | | | |

| **Levene's Test of Equality of Error Variances^a^** | | | |
| --- | --- | --- | --- |
| Dependent Variable: PETscore | | | |
| F | df1 | df2 | Sig. |
| 1.132 | 1 | 73 | .291 |
| Tests the null hypothesis that the error variance of the dependent variable is equal across groups. | | | |
| a. Design: Intercept + PSWQ + Affemp + COGEMP + Anx1Rel2 | | | |

## Assumptions for Regression Analysis


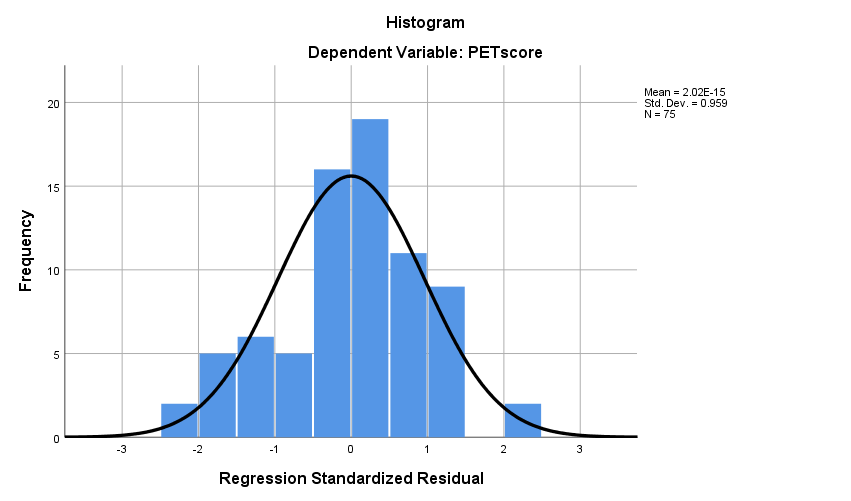


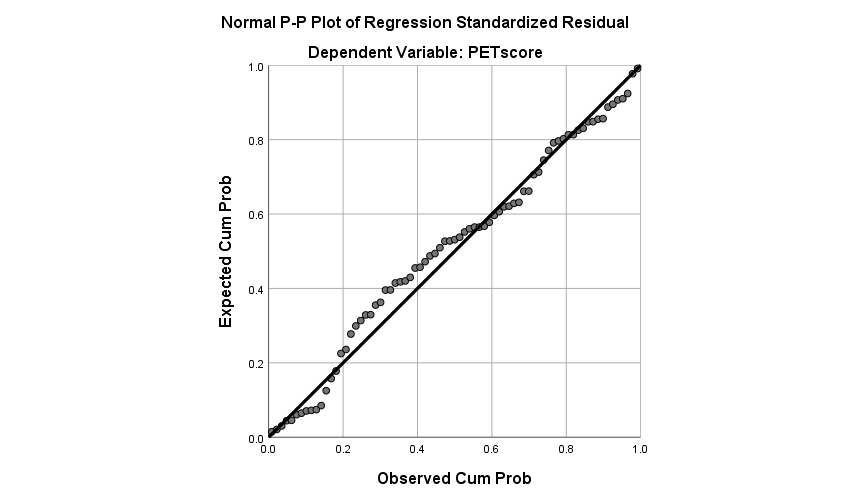


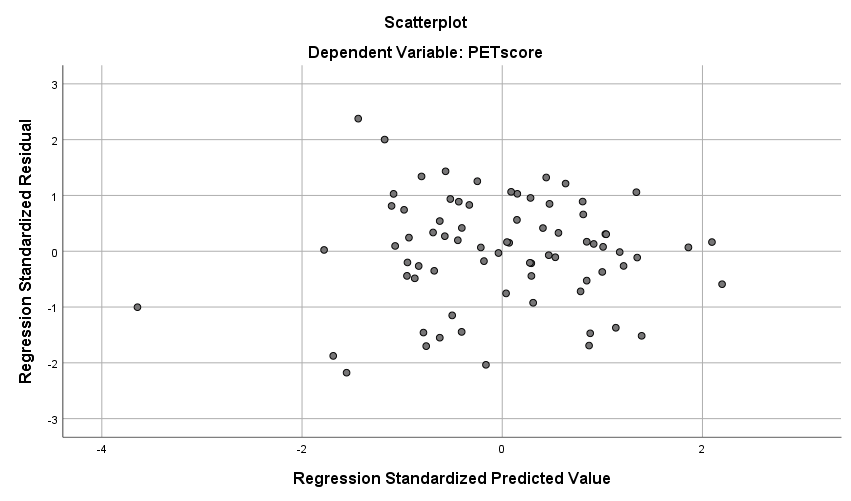


# Tables

**Table 1**

Correlations (Spearman’s Rho (p value)) between trait anxiety, trait empathy measures and state empathy measures

|  | **PSWQ** | **QCAE (Cognitive)** | **QCAE**  **(Affective)** | **RMET** | **PET** |
| --- | --- | --- | --- | --- | --- |
| **PSWQ**  **(Trait Anxiety)** | **-** |  |  |  |  |
|  | - |  |  |  |  |
| **QCAE**  **(Trait Cognitive Empathy)** | .213 | - |  |  |  |
|  | (.067) | - |  |  |  |
| **QCAE**  **(Trait Affective Empathy)** | .302^†^ | .360** | - |  |  |
|  | (.008) | (.001) | - |  |  |
| **RMET**  **(State Cognitive Empathy)** | -.260^†^ | .121 | -.115 | - |  |
|  | (.025) | (.151) | (.163) | - |  |
| **PET**  **(State Affective Empathy)** | .220 | .225* | .333** | -.144 | - |
|  | (.058) | (.026) | (.002) | (.109) | - |

***** *Correlation is significant at the 0.05 level (1-tailed).*

*** Correlation is significant at the 0.005 level (1-tailed).*

^†^ *Correlation is significant at the 0.05 level (2-tailed)*

^††^ *Correlation is significant at the 0.005 level (2-tailed)*

**Table 2**

Regression model predicting Affective Empathy with interaction terms

| **Variable** | ***B*** | ***SE B*** | ***β*** | ***Sig.*** |
| --- | --- | --- | --- | --- |
| Group (Anxiety/Relaxation) | .330 | .146 | .242 | .027 |
| Trait Anxiety | .009 | .010 | .147 | .346 |
| Trait Affective Empathy | .048 | .025 | .376 | .062 |
| Group x Trait affective empathy | .012 | .031 | .075 | .710 |
| Group x Trait Anxiety | -.013 | .014 | -.136 | .380 |
| Group x Trait Anxiety x Affective Empathy | .000056 | .001 | .005 | .967 |
